# What is the Role of Primary Prevention of Obesity in an Age of Effective Pharmaceuticals?

**DOI:** 10.1007/s13679-025-00632-0

**Published:** 2025-05-07

**Authors:** María Gómez-Martín, Oliver J. Canfell, Li Kheng Chai, Anna K. Jansson, Robyn Littlewood, Clair Sullivan, Dawn Power, Erin D. Clarke, Louisa Ells, Nienke De Vlieger, Tracy L. Burrows, Clare E. Collins

**Affiliations:** 1 School of Health Sciences, College of Health, Medicine and Wellbeing, The University of Newcastle, Callaghan, NSW 2308 Australia; 2 Food and Nutrition Research Program, Hunter Medical Research Institute, New Lambton Heights, NSW 2305 Australia; 3 Department of Nutritional Sciences, School of Life Course and Population Sciences, Faculty of Life Sciences & Medicine, King’s College London, London, UK; 4 Health and Wellbeing Queensland, Queensland Government, Brisbane, QLD Australia; 5 Queensland Digital Health Centre, Centre for Health Services Research, The University of Queensland, Saint Lucia, QLD Australia; 6 Metro North Hospital and Health Service, Queensland Health, Herston, QLD Australia; 7 School of Health, Obesity Institute, Leeds Beckett University, Leeds, UK; 8 School of Environmental and Life Sciences, College of Engineering, Science and Environment, The University of Newcastle, Ourimbah, NSW 2258 Australia

**Keywords:** Obesity, Primary prevention, Anti-obesity medication, Glucagon-like peptide-1 agonists, Clinical obesity, GLP-1/GIP, Diet, Nutrition

## Abstract

**Purpose of review:**

To examine the evidence and continuing role of strategies for the primary prevention and treatment of obesity in the context of effective obesity pharmacotherapies, through a narrative review.

**Recent findings:**

Global policies to improve nutritional labelling and reduce sugar-sweetened beverages consumption have been implemented worldwide (> 45 countries) with some success which varies by population and environment. Tailored behavioural interventions are effective and essential to reduce individual risk of progression from preclinical to clinical obesity. Pharmacotherapies are powerful treatment agents for clinical obesity but must consider nutritional and metabolic risks of use and discontinuation. The obesogenic environment continues to undermine individual agency to adopt healthier dietary and physical activity patterns. Population health informatics tools could inform tailored interventions based on real-time risk and contribute to obesity prevention and treatment.

**Summary:**

Efforts to rebalance investment towards obesity prevention must continue to improve population health and reduce healthcare burden.

**Supplementary Information:**

The online version contains supplementary material available at 10.1007/s13679-025-00632-0.

## Introduction

Obesity is a complex and multifactorial disease characterised by excessive adiposity [1]. It is a global health priority affecting all populations due to its prevalence and adverse health impacts. The global costs are predicted to reach US$ 3 trillion per year by 2030 and more than US$ 18 trillion by 2060 [2].

Traditionally, obesity is diagnosed by calculating an individual’s body mass index (weight divided by height squared), however, this can lead to both underestimations and overestimations of body fat in some population groups and provide inadequate information about an individual’s health. Consequently, this can weaken the effectiveness of medical care and health policies [1, 3].

Recently, The Lancet Commission on Clinical Obesity established criteria for the diagnosis of preclinical and clinical obesity. To overcome existing limitations in its definition, clinical obesity is now defined as a chronic illness characterised by alterations in the function of tissues, organs, the entire individual due to excess adiposity, which can lead to life-altering or life-threatening complications [3]. In contrast, preclinical obesity is defined as excess adiposity with preserved organ and tissue function, accompanied by an increased risk of progression to clinical obesity or other non-communicable diseases [3]. For individuals with preclinical obesity, healthcare needs to focus on risk reduction, primary (aimed at decreasing the number of new cases of clinical obesity) and secondary prevention (aimed at reducing the rate of established cases of clinical obesity) of clinical obesity and other obesity-related diseases.

While environmental changes have undoubtedly contributed to the rapid rise in pre- and clinical obesity prevalence, interactions between environmental, genetic and biological factors have also contributed [4]. Genetics contributes to substantial variation in body weight among individuals and determines their response to an ‘obesogenic’ environment [5, 6]. In response to this public health challenge, many governments have invested in primary prevention policies aimed at reducing the incidence of obesity [7]. Although prevention through education and changes to ‘obesogenic’ environments are long-term goals, early intervention and treatment to improve weight-related health is required for individuals with preclinical, and clinical obesity. Behavioural interventions (e.g., diet, physical activity, reduce sedentary behaviour, sleep) are first line treatment to both preclinical and clinical obesity and could include complementary therapies when necessary, such as bariatric surgery or pharmacotherapy that influence appetite [8, 9]. In recent years, there has been rapid progression in pharmacotherapies, including those initially designed for treatment of diabetes now showing efficacy in the treatment of obesity and obesity-related diseases [10–13]. Obesity is strongly linked to social position, local environment, and ethnicity and effectiveness of pharmacotherapies outside of controlled trial settings is likely mediated by these factors. Black African and Caribbean populations who received liraglutide lost significantly less weight and had greater attrition than patients of white ethnicity in a real-world NHS evaluation in the UK [14]. This suggests that individual response may be sensitive to mechanisms that are unique to real-world interventions, such as higher levels of deprivation, weaker financial position and agency, and lower nutrition-related knowledge and access, all of which require upstream policies and interventions to address. Primary prevention for obesity is essential to target structural socioeconomic and environmental determinants of obesity that pharmaceuticals cannot address.

In this narrative review, we adopt Rose’s framework [15] to synthesise the evidence for primary prevention and treatment of obesity in the context of effective pharmacotherapies. First, the population strategy, which adopts primordial and primary prevention approaches to lower and favourably redistribute the mean level of population risk [15]. A summary of major global policies for obesity prevention is presented, along with an appraisal of their effectiveness. Second, the high-risk strategy that includes multidisciplinary and multicomponent therapeutic interventions to treat and manage obesity in individuals, reducing risk for clinical obesity and obesity-related diseases [3]. Here, we discuss behavioural interventions and pharmacotherapies, including glucagon-like peptide-1 and glucose-dependent insulinotropic polypeptide receptor agonist (GLP-1/GIP RA) medications (liraglutide, semaglutide and tirzepatide). We also critically outline advantages and disadvantages of these novel pharmacotherapies, including dietary considerations. Finally, we examine the ongoing need for both population and high-risk strategies to optimise adiposity-related health and wellbeing in adults, including contemporary population health informatics tools to precisely guide both strategies.

## Global Strategies for Obesity Prevention

### Review of Policy Frameworks on Obesity

**Fig. 1:**
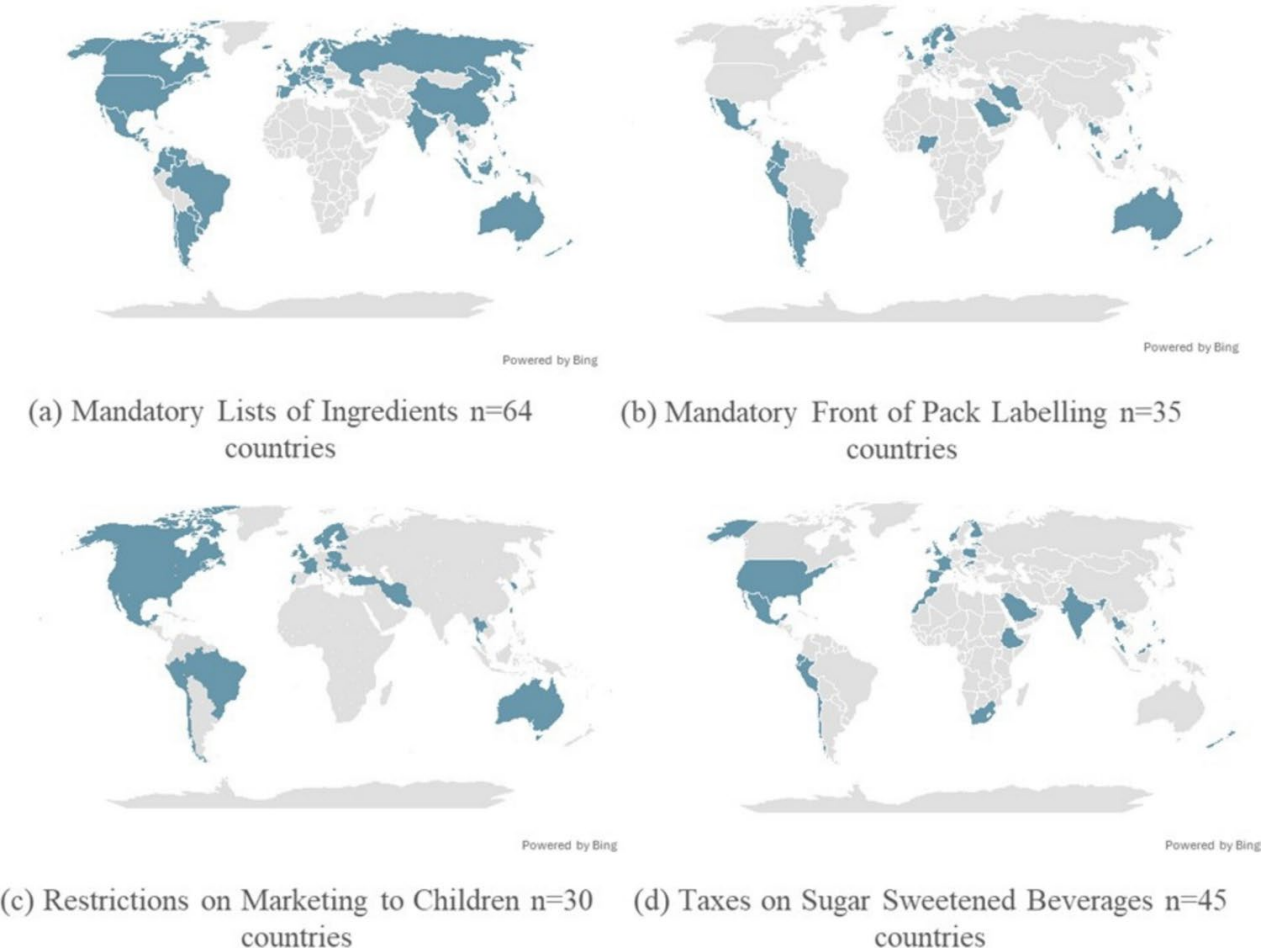
**a-d** Key policies implemented at the national level in various countries. Source: World Cancer Research Fund International’s NOURISHING database available at: https://policydatabase.wcrf.org/

Various global public health policy frameworks have been developed as prevention initiatives to address issues related to obesity, with a strong focus on nutrition and physical activity. The Commission on Ending Childhood Obesity (2016) [16] and the World Obesity Federation ROOTS framework (2020) [17] introduced a layered approach to influence health behaviour and reduce obesogenic environments. The World Cancer Research Fund (WCRF) developed the NOURISHING (2013) [18, 19] and later MOVING policy (2022) frameworks [20] to support governments in prevention efforts to promote healthier behaviours to prevent obesity and non-communicable diseases globally through policies, such as introduction of taxes on sugar-sweetened beverages (SSBs), front of pack labelling (FOPL), and food marketing restrictions. These frameworks emphasise the need for enhanced monitoring, surveillance, and a systems-based approach to obesity prevention, early intervention, and treatment across the life course. Figure 1 presents a selection of key policies that are more prominent and currently implemented on a national level in countries worldwide, highlighting the global effort in addressing obesity.

### Effectiveness Review of Policy Frameworks

FOPL first appeared in 1989 and had rapid uptake internationally [21], it was supported by the public and health professionals, but opposed by commercial interests [22]. Research suggests FOPL is likely to be cost-effective for addressing obesity by providing consumers with clearly visible and interpretative nutritional information to make informed choices, and encourages manufacturers to formulate/reformulate products to better meet health standards [23, 24]. Internationally, large variation exists with FOPL, however, the FOPL designs requiring least literacy or numeracy have greater impact on lower educated, lower income consumers [22].

Many countries over time have adopted and implemented policies focusing on reducing SSBs consumption for all ages [25]. Taxes on SSBs introduced in the early 2000s [26], are seen as an effective fiscal tool to promote health. Countries such as the UK, South Africa, and Mexico have introduced SSB taxes, which reduced consumption and generate funds for public health [25]. In the UK, manufacturers had reformulated products in the 2-year lead-up to the tax introduction [22]. In Mexico, SSB sales dropped by 5.5% in the first year and 9.7% in the second year, leading to a decline in childhood obesity rates [25]. Conversely, some countries have faced challenges implementing SSB taxes. For example, in South Africa, the sugar industry lobbied against the introduction of SSB tax; in one county in Illinois (USA), SSB tax was repealed after less than a year due to persistent public pressure fuelled by the SSB industry lobbying against the tax; and in Fiji, the SSB tax was reduced and revised after industry complaints about inconsistent taxation enforcement [27].

Research suggests that these policies are likely to be cost-effective, with the estimated implementation costs offset by the anticipated healthcare savings. The 2019 Organisation for Economic Co-operation and Development (OECD) analysis found that better food labelling is expected to bring significant impact in the short term, while restrictions on marketing to children for example reducing the times that these can be aired, offer the greatest impact long term, returning $6.6 saving for $1 invested [22, 28]. However, these may not be effective for some, given the individual differences in engaging with social media, television and similar platforms.

### Current Policies may not be Effective for Some Individuals

While cost-effective, FOPL and SSB taxes may not be effective for all populations groups as their impact varies across different environments and population groups. There may be many reasons for this, including modifiable (e.g., diet quality, physical activity levels), and non-modifiable factors (e.g., food availability and genetics) [29]. Geographic location, ethnicity and culture can also impact an individual’s risk of developing obesity and associated health conditions [30].

People living in rural and remote areas have an increased risk of poor health behaviours. This increased risk may be partially attributed to the food environment, a greater likelihood of engaging in poorer health-related behaviours (such as unhealthy diet and physical inactivity) and reduced access to health care services for preventative care [30, 31]. These communities require tailored prevention strategies that address the obesogenic factors associated with geographical remoteness, such as improving access to healthy foods and opportunities for physical activity by investing in accessible walking paths or providing community-based exercise programs [30]. This highlights the need for equity-focused efforts to achieve the highest level of health for all and reduce disparities in health and healthcare. One example of this could be co-designed health promotion programs that are championed by community members to ensure cultural relevance and improved engagement. These efforts ensure that prevention activities reach those most at risk, especially in rural and lower-income areas [32].

Similarly, socioeconomic status (SES) impacts an individual’s risk of living with obesity. Individuals with lower SES are more likely to live in environments with limited access to healthy foods, experience reduced food security and have lower nutrition literacy [33]. For example, public health policies could be focus on improving food environment by providing subsides for fresh produce in lower SES areas or by supporting community nutrition education programs that are culturally relevant and tailored to local needs [34]. However, even if food environments were universally improved, the impact of national policies at the individual level would be variable. Even in the presence of a supportive policy, many individuals will still require treatment to optimise weight-related health and prevent premature morbidity and mortality and facilitate secondary prevention.

## Obesity Treatment

### Behavioural Interventions

Behavioural interventions remain the cornerstones of treatment for both preclinical and clinical obesity treatment and could include individualised care plans and group programs that support health-related behaviour change (e.g., diet, physical activity, sedentary behaviour and sleep) and adjunctive therapies when needed, such as psychological, pharmacological and surgical [12, 35]. Studies such as WRAP [36] (Weight loss Referrals for Adults in Primary care) and DiRECT [37] (Diabetes Remission Clinical Trial) have demonstrated the efficacy of lifestyle behaviour change interventions (diet and exercise) in reducing adiposity. Medical Nutrition Therapy (MNT) is an effective dietitian-delivered nutrition treatment approach, personalised to meet an individual’s values, preferences and goals [38]. The use of MNT in clinical obesity treatment has been supported by position statements by the Academy of Nutrition and Dietetics and the Canadian obesity guidelines [38, 39]. Individualised dietary interventions can result in an additional 0.96 kg weight-reduction over 6 months’ time-period, compared to minimal or no intervention [40]. MNT can include a range of dietary interventions ranging from personalised approaches, focusing on nutritious healthy food, and optimising diet quality to specific dietary intervention with energy intake targets. In addition, advances in dietary interventions, including ‘omics’ technologies (i.e., nutrigenomics, metagenomics, and metabolomics), have potential to optimise personalised dietary support through MNT. For many individuals currently, pharmacotherapy or bariatric surgery, supported by MNT are needed to improve adiposity related health [12, 41, 42].

#### Very Low Energy Diets (VLEDs) and Low Energy Diets (LEDs)

Some examples of intervention with energy intake targets are the VLEDs and LEDs. VLEDs use formulated nutritionally complete meal replacement products to facilitate rapid weight loss (WL), providing approximately 600 kcal/day, the remainder of the intake is usually consumed at the patient's discretion. Meanwhile LEDs provide between 800–1200 kcal/day [43]. WL with these dietary approaches during the first 4 to 6 weeks could be up to 2.5 kg/week. However, evidence demonstrates WL reduces to approximately 0.8 kg/week after the initial 6 weeks and maintained thereafter in longer-term intervention trials in which VLEDs were tested for up to 6 months [43, 44]. It is important to note that sustaining a reduction in energy intake over a long period can be challenging, and the WL could be linked to loss of lean body mass which can have negative health implications [43].

#### Physical Activity/Reducing Sedentary Behaviour

A meta-analysis found a significant association between obesity and sedentary behaviour (OR 1.45, 95% CI, 1.21–1.75) and physical inactivity (OR 1.52, 95% CI, 1.23–1.87) [45]. Reducing sedentary behaviour and increased physical activity can be a useful strategy to prevent and manage obesity. For meaningful weight and total adiposity loss, at least 300–420 min of moderate intensity aerobic physical activity is recommended per week [46]. To prevent weight and adiposity gain, more than 150 and preferably 300 min per week of moderate intensity is required [46].

#### Sleep

Given the high rates of sleep abnormalities in those with increased weight, behavioural interventions are more frequently beginning to incorporate educational components on sleep. Existing research highlights the importance of not only getting enough hours of sleep but also other key aspects such as sleep hygiene, sleep latency, and sleep quality [47, 48].

### Pharmacotherapies

The first-generation medications that remain approved include orlistat (U.S. Food and Drug Administration (FDA) approval 1999) and naltrexone-bupropion (FDA approval 2014), showed promising effects for WL [49, 50]. However, due to their side effects, such as inhibition of fat absorption leading to faecal incontinence (orlistat), and nausea and constipation (naltrexone-bupropion), these treatments may not be suitable to everyone [51, 52].

### Glucagon-like Peptide-1 and Glucose-dependent Insulinotropic Polypeptide Receptors Agonist (GLP-1/GIP RA) Medication Era

New medications targeting the incretin system, have been developed for treatment of Type 2 Diabetes (T2D) and clinical obesity [53]: the GLP-1 and GIP RAs [54, 55]. The three major pharmacotherapies with current regulatory approval and recommendation for clinical use are liraglutide, semaglutide and tirzepatide (Table 1).

**Table 1:** Summary of the three major GLP-1/GIP receptor agonist class medications currently approved and available (liraglutide, semaglutide and tirzepatide)

Medication	Prescription and frequency of the dose	FDA approval		Receptor agonist	Mechanism of action	Reference
*Liraglutide*	Obesity (*Saxenda®*) and T2D (*Victoza®)* Daily	*Saxenda®* in 2014		GLP-1	Promoting insulin secretion, suppressing glucagon release, and reducing appetite These effects help improve glycemic control and contribute to significant weight loss	[56, 57, 11]
*Semaglutide*	Obesity (*Wegovy®*) and T2D (*Ozempic®)* Weekly		*Wegovy®* in 2021			[58–61]
*Tirzepatide*	Obesity (*Zepbound®*) and T2D (*Mounjaro®)* Weekly	*Zepbound®* in 2023		A novel dual GLP-1 and GIP	GLP-1 enhances insulin secretion, inhibits glucagon release, and reduces gastric emptying, all of which contribute to lower blood glucose levels and reduced hunger. GIP, while traditionally less studied, is known to enhance insulin secretion in response to meals Tirzepatide’s dual mechanism is thought to synergistically improve insulin sensitivity and promote weight loss through appetite suppression, decrease food intake and metabolic function	[62–64]

GIP: glucose-dependent insulinotropic polypeptide. GLP-1: glucagon-like peptide-1. T2D: Type 2 Diabetes

### Treatment Evidence and Comparison

We previously outlined the effectiveness of behavioural interventions and first-generation medications for clinical obesity treatment. This section will focus on the efficacy of GLP-1/GIP RA class medications.

Liraglutide has been studied extensively in the *SCALE* (2015) (Satiety and Clinical Adiposity–Liraglutide Evidence) trial program [11, 65]. In this trial, individuals treated with liraglutide 3.0 mg daily achieved a mean WL of 8.0% over 56 weeks, compared to a 2.6% WL with placebo (Table S1) [11]. On the other hand, semaglutide’s efficacy in obesity treatment was investigated in the *STEP* (Semaglutide Treatment Effect in People with obesity) trial series (2021). The *STEP-1* trial found that participants receiving semaglutide 2.4 mg weekly achieved an average WL of 14.9% over 68 weeks, compared to 2.4% in the placebo group [57]. This trial demonstrated semaglutide’s ability to significantly reduce body weight and improve related comorbidities, such as blood pressure and cholesterol levels. Additional trials further supported the efficacy of semaglutide in various populations, including those with T2D, and demonstrated its superiority over liraglutide (STEP-8) (Table S1) [66–69]. The efficacy of tirzepatide for obesity was evaluated in the *SURPASS* (2021) and *SURMOUNT* (2022) clinical trial programs [10, 70]. Tirzepatide demonstrated superior WL compared to a placebo. Participants treated with tirzepatide (5, 10, and 15 mg doses) achieved an average WL of up to 20.9%, with the highest dose showing the most pronounced effects [10]. Tirzepatide also led to significant improvements in metabolic parameters, including glycaemic control and cardiovascular risk factors.

Eli Lilly and Company has released the data for the trial SURMOUNT-5 (2024), observing that on average, tirzepatide led to a superior WL of 20.2% compared to 13.7% with semaglutide (press note) [71].

These new medications represent significant advancements in the pharmacological management of clinical obesity. Tirzepatide’s dual action on GLP-1 and GIP receptors offers a novel approach with exceptional efficacy in promoting WL. Semaglutide, with its potent GLP-1 RA properties, has already become a cornerstone treatment for obesity, showing impressive WL outcomes in clinical trials [57]. Liraglutide, while less effective than its newer counterparts, remains an important option for patients with obesity, particularly in combination with behaviour changing interventions. The clinical data supporting these agents highlight their transformative potential for improving long-term weight management and reducing obesity-related comorbidities, providing new hope for individuals struggling with this chronic condition.

## Disadvantages of GLP-1/GIP RA Class Medications

Despite their effectiveness for WL, GLP-1/GIP RA class medications are not without limitations. At one year post withdrawal of semaglutide, two-thirds of weight lost is regained and cardiovascular disease (CVD) risk factors return to baseline [72], suggesting ongoing treatment and monitoring is required to maintain improvements, or better guidance on initiation of medication is required. Additional drawbacks include physical side effects, high costs, inequitable access [73], and the uncertainty surrounding their long-term health impacts [74]. Approximately 1 in 10 individuals taking GLP-1/GIP RA class medications experience gastrointestinal implications including nausea, vomiting, diarrhoea, constipation and colonic ischemia [75, 76]. These symptoms can lead to dehydration and other serious health complications such as kidney damage [75]. Additionally, recent research observed that GLP-1 medication increased the risk of hypotension, syncope, arthritic disorders, nephrolithiasis, interstitial nephritis and drug-induced pancreatitis [77]. Long-term side effects are not fully understood in specific population groups, which is an important consideration for those commencing treatment during childhood and adolescence. There is also conflicting evidence regarding impacts on bone [78] and muscle health [79], and a need for evidence on the potential exacerbation or development of vulnerable groups with increased risk of disordered eating pathologies [80], and other risks to mental health outcomes, encompassing depression, anxiety, and suicidal behaviour [81].

People living with preclinical, clinical obesity and diabetes have a higher prevalence of sarcopenia [82] and can face a double burden of malnutrition [83], which warrants attention. Furthermore, without adequate nutritional support during treatment initiation of GLP-1/GIP RA class medications, these risks may be amplified a concern that remains underexplored, as clinicians often lack sufficient training in nutrition and weight management [84]. A further concern is the predominance of privately funded treatment and the emergence of a black market for medication distribution and counterfeit products. This can lead to inequitable and financially prohibitive access [85], and severe consequences associated with unregulated and inappropriate use. The impact of losing access to medications can also result in psychological distress, and the absence of sustained WL can exacerbate feelings of failure and reduce motivation for behaviour change [86].

Given these uncertainties, further research into the long-term health implications is urgently needed. There is also a pressing need for policies to address black market sales of medications, and clear guidelines that ensure equitable access and person-centred care that incorporates appropriate prescription practices, nutrition support, behaviour change interventions, and psychological support that is tailored to need.

## Considerations for the Current and Future Pharmaceutical Era

### Dietary Considerations

Dietary considerations in the pharmacological literature vary greatly and reporting is limited. Trials such as STEP and SURMOUNT in adults include dietary counselling, which prescribes 500 kcal/day reduction as part of the dietary intervention [10, 57]. Although, there is a limited focus on improving dietary quality as part of the dietary interventions. The effects of GLP-1/GIP RA class medications on dietary intake and diet quality are not fully understood. A review of 10 studies found reduced energy intake in most studies, but only 4 examined macronutrient changes, highlighting the need for further research [87]. Therefore, it remains unclear whether individuals who are prescribed these medications adopt healthier behaviours, that may have contributed to the root causes of their clinical obesity. GLP-1/GIP RA class medications are powerful appetite suppressants and with fewer calories consumed, the focus on macro and micronutrients intake becomes even more important, to avoid developing protein-energy malnutrition, nutrient deficiencies and a potential acceleration of bone loss and mental ill health, further aggravated by insufficient vitamin D levels and inadequate calcium intake [87, 88].

GLP-1/GIP RA class medications also have several side effects, particularly during the up-titration phase, which can last for up to 20 weeks. Many of these side effects can be modified using dietary interventions [88], emphasising further the importance of dietary consideration during GLP-1/GIP RA treatment.

#### The Continued Need for Primary and Secondary (Clinical) Prevention

### Consequences of Obesity on Health and Wellbeing

People with preclinical obesity are at a significantly higher risk of developing chronic illnesses, such as clinical obesity, CVD, T2D and some cancers like colorectal or postmenopausal breast cancer [3, 89]. While reduction in adiposity alone can reduce these risks and improve overall health, the adoption of sustainable behaviour change plays an equally important role. Previous research has demonstrated the effectiveness of interventions including behaviour change strategies (e.g., education, identification of barriers/facilitators, goal setting) for improved long-term behaviour change and health risk reduction. For example, the Diabetes Prevention Programme for individuals with prediabetes demonstrated a 5–7% reduction in weight and was associated with a 58% lower risk of developing T2D [90, 91]. Creating better, more equitable interventions focused on behavioural change in individuals with preclinical obesity is therefore essential for the primary and secondary prevention of clinical obesity.

In addition, as key components of primary and secondary prevention strategies, behavioural interventions, not only promote weight reduction but also improve overall health, well-being and quality of life [92–94]. Beyond mitigating the risk of chronic diseases, these changes have been associated with additional benefits, including better sleep behaviours, improved mental health, metabolic health, and reduced inflammation [95–98], which in turn contribute to greater success in achieving and maintaining a healthy weight.

Therefore, both the emphasis on behavioural changes as part of the clinical prevention and treatment of obesity and on achieving a less obesogenic environment that facilitates these changes, remain important and should continue to be a priority for both public health and clinical treatment programs.

### Importance of Maintenance After Obesity Treatment

In the era of effective clinical obesity pharmacotherapies, there is a need for research on interventions following initial treatment phase of more recent medications. Although ongoing studies suggest that pharmacotherapy alone could potentially reduce the prevalence of clinical obesity, there is currently insufficient evidence to support its long-term effectiveness, due in part to the short time period for which they have been available. Existing and historical service delivery models treat obesity as an acute illness, often with short-term (2–12 months) behavioural interventions with limited (< 1 year) or no supported maintenance period [99]. However, the recent Lancet Commission on Clinical Obesity specified that individuals with clinical obesity should have timely access to comprehensive care and evidence-based treatments, as appropriate for individuals with a chronic disease [3]. The current break-fix healthcare models do not align with the chronicity of clinical obesity and must be augmented with accessible, sustainable, and effective post-treatment models of care to maintain the positive weight outcomes associated with GLP-1/GIP RA class medications. These maintenance models of care will require intensive, evidence-based behavioural therapy that already exists as the cornerstone of obesity treatment [100]. Care models can shift from using behavioural therapy for WL to optimising overall physical, nutritional and psychological health, especially following a period of lower calorie consumption [53].

Despite the increasing availability of effective pharmacotherapies, obesity prevalence internationally continues to follow a social gradient, with individuals living in socioeconomic disadvantage experiencing disproportionately worse prevalence [101], mortality [102] and cardiometabolic outcomes [103]. Clinical practice guidelines for weight maintenance post-treatment do not exist. Although some guidelines recommend combining diet, exercise and cognitive behavioural therapy to maintain weight loss, these recommendations are general and do not address the nutritional, psychological and cardiometabolic risks associated with medication discontinuation [104]. These risks include weight regain, resurgence of risk factors of cardiometabolic disease, reduced lean body mass, and nutritional deficiencies [72, 82, 83]. Weight remains sensitive to the social, environmental, commercial, and political determinants of dietary, physical activity, sleep and sedentary behaviours. Even with effective pharmacotherapies, models of care for obesity will continue to discharge individuals back to the environmental conditions that contributed to obesity. Efforts to rebalance investment and evidence generation towards primary prevention of preclinical and clinical obesity for communities and populations is imperative and must continue to ensure patients discharged after GLP-1/GIP treatment can return to an environment that supports weight maintenance and optimal physical and mental health.

### How Can Clinical Obesity Be Prevented in the Future?

Current health care systems are ill-equipped to reduce obesity rates and prevent future cases. Obesity prevention requires highly targeted interventions based on risk and need to make best use of limited financial and healthcare resources, regardless of global setting. ‘Proportionate universalism’ is a public health principle proposed by the UK Marmot Review [105] that extends Rose’s theory of prevention [15], whereby the scale and intensity of universal health services and interventions matches the degree of need within a target population [106]. This requires systematic identification of high-risk subgroups and continuous monitoring of outcomes that can be achieved with digital technologies. Novel digital tools to enable highly targeted interventions are emerging in high-income settings. The USA (RiskScape), Canada (PopHR), and Australia (PopHQ) are leveraging real-world data from electronic medical records to monitor obesity and chronic disease prevalence and risk factors, including social, biomedical, genomics, and environmental factors, in near real-time at a population level [107–111]. Integrating these population health informatics tools into routine public health practice can help to tailor service provision and interventions to the severity of need over time, place and person. Contemporary examples include population dietary surveillance using supermarket purchasing data, leveraging big data to monitor BMI trajectories in children [112], developing prediction models for obesity risk [113–115], and genomics-based screening and intervention across the life course [116, 117]. Development of digital surveillance tools and risk prediction models is common, however evidence supporting their application to improve obesity policy or practice is less clear and needs to be generated [108].

In practice, readiness for data-driven public health, or so-called ‘digital public health’ or ‘precision public health’, is highly dependent on micro-factors (individual systems and users), meso-factors (systems to collect data), and macro-factors (data re-use at scale) [118]. These factors are highly variable between local settings and countries, and low-middle income countries (LMICs) tend to have less core digital infrastructure, such as electronic health records, difficulties with data accuracy, completeness, and accessibility, and so overall lower use of health data than high-income countries [119]. In LMIC settings, health system fragility and cost barriers can impede capacity to deliver the right intervention at the right time [120]. Out-of-pocket payments cover almost half of the total health care costs in LMICs, compared to 30% and 14% in middle-income and high-income countries, respectively [120]. In the context of obesity prevention in LMICs, interventions outside the healthcare setting can focus on early life risk factors to improve health behaviours and prevent future obesity. School-based interventions for obesity prevention in LMICs mostly have positive effects on adiposity-related outcomes [121], dietary behaviour and physical activity [122]. Additionally, the World Health Organisation has identified highly cost-effective policies to reduce obesity, or ‘best buys’, featuring mass media campaigns promoting healthy diet and physical activity, and reformulating products to reduce salt and replace trans-fat with polyunsaturated fat [123]. A recent review of research in LMICs to evaluate these policies only found two studies focused on physical activity and diet in Southern India and Pakistan [123]. Recent efforts to digitise health care systems in LMICs [119], such as India’s Global Initiative on Digital Health, will accelerate ability to develop data-driven strategies for population-level obesity prevention [124].

In addition to proportionate and cost-effective population-level interventions, meaningful progress in obesity prevention is contingent upon health system reform. Transitioning from a reactive break-fix healthcare model to a proactive predict-prevent model is likely to improve healthcare sustainability and cost-effectiveness. Many global regions, including countries in the Americas (USA, Canada), Europe (Germany, Norway, Switzerland, Finland, France, Netherlands), and Asia–Pacific (Australia) structure their health care services with activity-based funding, a system where individual episodes of care are counted as activity and financially compensated. This financial model rewards disease-based break-fix care to the detriment of health-based predict-prevent care, as it is more difficult to count and measure prevention outcomes [125]. To successfully integrate prevention interventions into existing health care systems and public health policies, primary prevention of obesity needs to be measured such that it ‘matters’[125]. In high-income settings, this may be enabled through routinely collected data and informatics via linked health care and administrative datasets, beginning from the first 1,000 days. In LMICs, counting obesity prevention requires prioritising testing and evaluating ‘best buy’ interventions in healthcare and public health with traditional research infrastructure, and gradually incorporating digital tools as maturity progresses over time. Ultimately, modern primary prevention of obesity will advance with continued action on health inequalities across the life course, sound investment in cost-effective ‘best buy’ policies and early life interventions, enabled by digital transformation of the prevention and public health sector.

This narrative review provides a timely overview of emerging evidence on GLP-1/GIP RA class medications use, highlighting key behavioural interventions, population-specific considerations and the need for robust primary prevention. We have identified areas where further research is needed including systematic reviews to explore specific sub-topics in greater depth and guide targeted, evidence-based interventions. The current review took a narrative approach and therefore limitations include no formal critical appraisal or risk of bias assessment was undertaken as well as providing selective coverage of topics based on expert author consensus.

## Conclusion

Population-wide approaches to obesity prevention are critical for addressing the “upstream” causes of obesity and reducing reliance on pharmacotherapies. However, given the high global prevalence of obesity and the advent of highly effective and cost-effective pharmacotherapies, both population prevention and individual treatment strategies are necessary to reduce obesity prevalence and related costs. Both strategies must be prioritized to effectively manage obesity and its associated chronic diseases.

Internationally, policies have been created to improve food labelling and reduce SSB consumption in over 45 countries, with varying success. Tailored behavioural interventions are essential to prevent progression from preclinical to clinical obesity. While pharmacotherapies are effective for clinical obesity, their nutritional and metabolic risks must be considered. The obesogenic environment undermines individual efforts to adopt healthier lifestyles. Population health informatics tools that draw upon real-world data, like electronic health records, can support tailored interventions and contribute to obesity prevention and treatment.

In conclusion, behavioural interventions are central components for treating preclinical and clinical obesity and should include individualised care plans and adjunctive therapies, such as pharmacological, when needed. The use of new GLP-1/GIP RA class medications has increased due to their effectiveness but they come with side effects. Without major public health strategies to improve nutrition and physical activity that address the obesogenic environment, individuals will remain susceptible to positive energy imbalance. While existing obesity prevention efforts show promise, future obesity prevention initiatives could utilise digital tools to address both prevention and treatment of preclinical and clinical obesity through tailored, person-centred approach. Efforts to rebalance investment towards obesity prevention must continue to improve population health with a goal to reduce the healthcare burden for all.

## Key References

- Rubino F, Cummings DE, Eckel RH, Cohen RV, Wilding JPH, Brown WA, et al. Definition and diagnostic criteria of clinical obesity. Lancet Diabetes Endocrinol. 2025. 10.1016/S2213-8587(24)00316-4.
  - Lancet Commission has established criteria for the diagnosis of clinical obesity. This recent definition represents a consensus from leading international experts in the field and updates previous definitions, incorporating metabolic and clinical factors beyond BMI alone.
- Walmsley R, Sumithran P. Current and emerging medications for the management of obesity in adults. Med J Aust. 2023;218(6):276–83. 10.5694/mja2.51871.
  - This review provides a summary of existing and upcoming medications for managing obesity. It offers a timely perspective on available medications, including their mechanisms of action, efficacy, and clinical applications.
- Wilding JPH, Batterham RL, Calanna S, Davies M, Van Gaal LF, Lingvay I, et al. Once-weekly semaglutide in adults with overweight or obesity. N Engl J Med. 2021;384(11):989–1002. 10.1056/NEJMoa2032183.
  - This is large-scale, randomised controlled trial (level 1 evidence) provides robust evidence on semaglutide impact on weight reduction and its clinical relevance. Given the growing role of GLP-1 receptor agonists in obesity management, this study serves as a key reference supporting the effectiveness of semaglutide as a pharmacological treatment.
- Aronne LJ, Sattar N, Horn DB, Bays HE, Wharton S, Lin WY, et al. Continued treatment with tirzepatide for maintenance of weight reduction in adults with obesity: The SURMOUNT-4 Randomized Clinical Trial. JAMA. 2024;331(1):38–48. 10.1001/jama.2023.24945.
  - This is a recent large-scale trial, randomised, demonstrating the evidence of tirzepatide for weight loss in more than 600 participants and serves as a key reference in validating tirzepatide as a pharmacological treatment.

## Supplementary Information

Below is the link to the electronic supplementary material.

Supplementary file1 (DOCX 48 KB)

## Data Availability

No datasets were generated or analysed during the current study.
