# Supplementary material for: What is the Role of Primary Prevention of Obesity in an Age of Effective Pharmaceuticals?
Source: Curr Obes Rep. 2025 May 7;14(1):39. doi: 10.1007/s13679-025-00632-0 (PMC12058953; doi:10.1007/s13679-025-00632-0)
Supplement: Supplementary file 1 — Supplementary file1 (DOCX 48 KB) [file 13679_2025_632_MOESM1_ESM.docx]

**Supplementary material**

**Title:** What is the role of primary prevention of obesity in an age of effective pharmaceuticals?

**Authors:** María Gómez-Martín^1,2^, Oliver J Canfell^3^, Li Kheng Chai^4^, Anna K Jansson^1,2^, Robyn Littlewood^4^, Clair Sullivan^5,6^, Dawn Power^7^, Erin D Clarke^1,2^, Louisa Ells^7^, Nienke De Vlieger^2,8^, Tracy L Burrows^1,2^, Clare E Collins^1,2*^

**Authors name:**

María Gómez-Martín, ^1,2^ PhD

Oliver J Canfell, ^3^ RD, APD, PhD

Li Kheng Chai, ^4^ PhD

Anna K Jansson, ^1,2^ BPsyc (Hons), PhD

Robyn Littlewood, ^4^ FDA, PhD

Clair Sullivan, ^5,6^ MBBS (Hons) MD

Dawn Power, ^7^ BSc (Hons), PG Dip, RD, PhD candidate

Erin D Clarke,^1,2^ APD, PhD

Louisa Ells, ^7^ BSc (Hons), PhD, RNutr (public health), FRSPH

Nienke De Vlieger, ^2,8^ PhD

Tracy L Burrows, ^1,2^ APD, PhD

Clare E Collins, ^1,2^ FDA, PhD

**Author Affiliations:**

1. School of Health Sciences, College of Health, Medicine and Wellbeing, The University of Newcastle, Callaghan, NSW, 2308 Australia.
2. Food and Nutrition Research Program, Hunter Medical Research Institute, New Lambton Heights, NSW, 2305 Australia.
3. Department of Nutritional Sciences, School of Life Course and Population Sciences, Faculty of Life Sciences & Medicine, King's College London, London, UK
4. Health and Wellbeing Queensland, Queensland Government, Brisbane, Queensland, Australia.
5. Queensland Digital Health Centre, Centre for Health Services Research, The University of Queensland, Saint Lucia, Queensland, Australia.
6. Metro North Hospital and Health Service, Queensland Health, Herston, Queensland, Australia
7. School of Health, Obesity Institute, Leeds Beckett University, Leeds, UK.
8. School of Environmental and Life Sciences, College of Engineering, Science and Environment, The University of Newcastle, Ourimbah, NSW, 2258, Australia.

***Corresponding author:**

Clare E Collins

Advanced Technology Centre, University of Newcastle,  University Drive, Callaghan,  NSW 2308, Australia

*Email:* clare.collins@newcastle.edu.au  *Phone:* (02) 4921 5646 *Fax:* (02) 4921 7053

**Table S1.** Summary of examples of some of the main randomised controlled trials of behavioural interventions for weight loss or GLP-1/GIP receptor agonist medications.

| **Study** | **Participants** | **Study length** | **Interventions** | **Mean weight loss (% or kg) at primary time point** | **Estimated treatment difference** |
| --- | --- | --- | --- | --- | --- |
| ***Behavioural*** |  |  |  |  |  |
|  |  |  |  |  |  |
| WRAP [32] | 1,269 participants; 68% women, mean age 53years; mean BMI 34.5 kg/m² | ***Intervention***  12 weeks  52 weeks  ***Maintenance:***  Follow-up over 2 years. | Brief advice and self-help materials, a weight-management programme (Weight Watchers) for 12 weeks, or the same programme for 52 weeks. Primary outcome weight at 1 year of follow-up. | -4.75 kg 12 weeks and - 6.76 kg 52-weeks programme (control -3.26 kg) at 1 year of the follow-up | Behavioural program vs brief intervention: –2.71 kg (CI -3.86, -1,55) 52 vs 12 weeks program: -2.14 kg (CI -3.05, -1.22). All *p*<.0001 |
| DiRECT [33] | 298 participants with T2D <6 years duration; 41% female, mean age 54 years; mean BMI 35 kg/m² | ***Intervention***  2 years  ***Maintenance:***  intervention group was offered continued low-intensity support | Withdrawal of medications for diabetes and hypertension; 825 to 853 kcal/day total diet replacement for 3-5 months then structured food reintroduction and maintenance; physical activity up to 15,000 steps/day; individual sessions every second week for 20 weeks, then monthly for 2 years | -7.6 kg (control -2.3 kg) | -5.3 kg (CI -6.7, -3.9, *p*<0.0001) |
| ***Pharmacotherapies*** | |  |  |  |  |
| **GLP-1/GIP RA** | |  |  |  |  |
| ***Liraglutide*** |  |  |  |  |  |
| SCALE [11] | 3,731 participants without T2D and had a BMI ≥30 or a BMI≥27 if they have DL or HT; 78.5% females; mean age 45.1 years mean BMI 38.3 kg/m^2^ | ***Intervention:***  56 weeks  ***Maintenance:***  12 weeks to assess safety and efficacy after drug discontinuation | Liraglutide (3.0 mg subcutaneous injection) or matching placebo. Both groups received 13 counselling on lifestyle modification* | Intervention:  -8.0%  Control:  -2.6% | Liraglutide −5.4% (CI −5.8,  -5.0; *p*<0.001) compared to placebo at 56 weeks. |
| SCALE-IBT [61] | 282 participants with a BMI ≥30; 83% females | ***Intervention:***  56 weeks  ***Maintenance:***  30-days safety follow up | Liraglutide (3.0mg subcutaneous injection) or matching placebo. Both groups received an Intensive Behavioural Therapy (23 sessions) and prescribed a low-calorie diet based on body weight. | Intervention:  -7.5%  Control:  -4.0% | Liraglutide-IBT -3.4% points (CI -5.3, -1.6, *p*<0.001) compared to placebo at 56 weeks. |
| ***Semaglutide*** |  |  |  |  |  |
| STEP-1 [54] | 1,961 participants  without diabetes, with a BMI ≥30 or BMI ≥27 plus a weight-related condition; 74% females, mean age 46 years, mean BMI 37.9 kg/m^2^ | ***Intervention:***  68 weeks  ***Maintenance:***  7 weeks off treatment follow-up | Semaglutide (2.4 mg subcutaneous injection) or matching placebo, in addition to lifestyle intervention* (18 counselling sessions). | Intervention:  -14.9%  Control: -2.4% | Semaglutide -12.4% points (CI -13.4,  -11.5; *p*<0.001) compared to placebo at 68 weeks. |
| STEP-3 [64] | 611 adults without diabetes, with a BMI ≥30 or BMI ≥27 plus a weight-related condition, and a history of unsuccessful dietary weight loss attempts.  81.0% females, mean age 46 years, mean BMI 38.0 kg/m^2^ | ***Intervention:***  68 weeks  ***Maintenance:***  7 weeks off treatment follow-up | Semaglutide (2.4 mg subcutaneous injection) or matching placebo, both groups received a low-calorie diet** for the first 8 weeks and intensive behavioural therapy (30 counselling sessions). | -16.0% (control -5.7%) | Semaglutide  -10.3% points  (CI -12.0, -8.6; *p*<.001) compared to placebo at 68 weeks |
| STEP-8 [62] | 338 adults  without diabetes, with a BMI ≥30 or BMI ≥27 plus a weight-related condition. 78.4% females, mean age 49 years, mean BMI 37.5 kg/m^2^ | ***Intervention:***  68 weeks  ***Maintenance:***  7 weeks off treatment follow-up | Semaglutide (2.4 mg subcutaneous injection), Liraglutide (3.0mg subcutaneous injection) or matching placebo, plus lifestyle intervention* (15 counselling sessions). | Semaglutide:  -15.8% Liraglutide:  -6.4%  Placebo:  –1.9% | Semaglutide -9.4 % points (95% CI, -12.0 to -6.8; *p*<.001) compared to Liraglutide at 68 weeks. |
| SELECT [65] | 17,604 adults without T2D, ≥45 years, BMI≥ 27 and had established cardiovascular disease, 28% females, mean age 61.6 years, mean BMI 33.3 kg/m^2^ | Primary efficacy end point was a composite of death from cardiovascular causes, nonfatal myocardial infarction, or nonfatal stroke. | Semaglutide (2.4 mg subcutaneous injection) or matching placebo | Semaglutide:  -9.4%  Control:  -0.9% | Semaglutide -8.5% points (CI -8.8, -8.3) compared to placebo at 104 weeks. |
| ***Tirzepatide*** |  |  |  |  |  |
| SURMOUNT-1 [10] | 2,539 adults without T2D and with a BMI ≥30 or BMI≥27 and ≥1 weight-related condition, 67.5% females, mean age 44.9 years, mean BMI 38 kg/m^2^ | ***Intervention:***  72 weeks  ***Maintenance:***  4 weeks safety follow-up | Tirzepatide (5, 10, or 15 mg subcutaneous injection) or volume matched placebo plus lifestyle intervention* (20 counselling sessions). | Tirzepatide  5 mg: -15.0% Tirzepatide  10 mg: -19.5% Tirzepatide  15 mg: -20.9% Control:  −3.1% | Compared to placebo at 72 weeks:  Tirzepatide 5 mg: -11.9% points (CI -13.4, -10.4) Tirzepatide 10 mg:  -16.4% points (CI  -17.9, -14.8). Tirzepatide 15 mg, -17.9 % points (CI -19.3, -16.3), all *p*<0.001. |

*Lifestyle modification: Reduced-calorie diet (500 kcal/day deficit) and physical activity (150 mins/week). **Diet: 1000-1200 kcal/day for first 8 weeks, 1200-1800 kcal/day for rest of treatment and Physical activity: 100 mins/week increasing by 25 mins every 4 weeks to reach 200 min/week.
